# Collectanea

**Published:** 1833-08

**Authors:** 


### COLLECTANEA.

Floriferis ut apes in saltibus omnia libant,
Omnia nos, itidem, depascimur aurea dicta.

### PHYSIOLOGY.

*Case of Anencephalism.* A POOR woman, aged thirty, mother of two children, was delivered of a third in April, 1830; it was male and anencephalic, and yet it lived for thirty-two hours. Since that period, the woman had dreaded being again pregnant, as "she was certain (she said) that she should bear nothing but monsters." On 4th July of next year, she was delivered of a female child; and this also was anencephalic. It lived for twenty hours. The respirations were feeble and distressing, and, soon after birth, the face and body became of a blue colour, which deepened more and more to a blackish hue. Its size was that of a full-grown and healthy child. The shape of the head was most extraordinary; the level of its vertex was on a line with the root of the nose, its dimensions were much contracted, as well from side to side as from before backwards; the face was fully formed, but the forehead and eye-brows were wanting; the eyes were startingly projecting, in consequence of the deficiency of the upper part of their orbit, and were situated at the most elevated points of the head; the inclination of the face was sloping backwards, so that the facial angle was much smaller than usual. On a careful examination after death, the whole of the vault of the skull was found wanting, and the open cavity was bounded by a rim formed by the frontal, parietal, and occipital bones; the basis of the cranium was much contracted in size in all its dimensions; the posterior wall was inclined forwards, so that the os occipitis appeared to rise vertically, in a line with the margin of the foramen magnum, and hence there were, in reality, no occipital fossæ; the anterior fossæ were scarcely distinguishable; there was no trace of the cribriform plate of the ethmoid bone; the os frontis consisted of two small pieces, joined to each other in the median line; and each piece consisted of two laminæ, one of which represented the orbital plate, the other the globular portion; the foramen opticum of the sphenoid was wanting; the petrous process of the temporal was fully formed, while the squamous plate was very imperfect; the parietal bones, in shape and appearance, were like a narrow band or rim along the upper edge of the temporal bones; the basilar process was placed vertically. All the vertebræ were perfect. The scalp was covered with long hair, and extended somewhat beyond the bony margin of the cranium, and then became suddenly continuous with a red-coloured thin membrane, which supplied the place of the vertex; it adhered to the subjacent parts, and had much the appearance of a cicatrix. On dissecting this off, a soft tumor, of the size of a walnut, and of a fibro-cellular tissue, without any trace of cerebral substance, was found to occupy the central part of the cranium; it adhered strongly to the dura mater in all directions: the cerebrum, cerebellum, and tuber annulare were quite deficient; the upper end of the medulla oblongata was to be seen, and it presented the appearance of having been cut from the tuber; it had no connexion with the fibrous substance described above. The spinal marrow was also quite normal. There was no trace of the optic and olfactory nerves in the cranium: in the orbit, the former was found, but very small, flattened, and reduced to an empty neurilema; posteriorly it was lost in the dura mater; the eyes were large, and the retina perfectly developed. There was no trace of any of the cerebral nerves within the cranium, except of the tri emini, which appeared to proceed from a plexiform ganglion, situated in the fibrous mass; and, as this substance was infiltrated with blood, the nerves were with difficulty traced. The facial and auditory nerves were not attached to the medulla oblongata, but lay loose and unattached in the cavity of the cranium; on the outside of the cranium they seemed quite healthy; the auditory organs were fully formed. The spinal nerves were all normal in structure and arrangement. The carotid arteries were very small, and terminated in the fibrous mass; the vertebral were shrivelled to mere threads. Lungs nearly healthy; the ductus arteriosus and the foramen ovale were quite open. The abdominal viscera were perfect; meconium was found in the large intestines.—*Med. Chir. Journal.*

---

### PATHOLOGY.

*Cases of Dropsy and Gangrene occurring in a Family who had subsisted for some time on unwholesome Potatoes; with Remarks.* By Alexander Peddie, Esq. Surgical Hospital.

Case I. On the 13th December 1832, I was requested to visit Mary Clapperton, ætat. nineteen months, at the Causewayside, as an out-door patient of the Edinburgh Surgical Hospital. On her left cheek, half way between the mouth and angle of the jaw, there was a portion of integument of the size of a half-crown piece, dark, pulpy, and exhaling a fetid smell. Over the cheek and the greater part of the anterior surface of the neck the parts were much swelled, very hard, and of a deep red colour; some parts, especially in the immediate vicinity of the sore, having a yellowish tinge, and others a bluish black appearance. The child had a dull listless aspect, a loaded tongue, and a quick feeble pulse.

The parents stated, that a few weeks before, she had had a mild attack of scarlet fever, the rash of which extended merely over the chest, and receded in the course of a day. A small hard swelling then appeared a little below the present sphacelated part, and after some blood was abstracted from it by leeching, the appearances just described were gradually displayed. On questioning the parents as to the kind of food used by the patient, they assured me that it was perfectly wholesome.

I immediately removed the mortified part, and found a considerable cavity underneath, and the integuments undermined in every direction, apparently extending nearly to the limits of the external appearances already described. The Unguentum Resinosum with an equal part of the Oleum Terebinthinæ, covered by a poultice, was applied to the sore; and this dressing was removed four times a day. I directed also that a purgative should be taken immediately, and that the child's strength should be supported by a liberal allowance of wine. In consequence of the above dressings to the sore, the application of the Tinctura Saponis c. Opio to the surrounding parts, and the exhibition of wine, of which about one gill was taken daily, in a few days the greater bulk of the swelling had diminished, the parts assumed a healthy colour, the ulcer showed a good healing surface, and the patient appeared lively. The wine was now withdrawn; sago provided as diet; and a lotion of the sulphate of zinc, with a compress and bandage, applied to the parts. Under this treatment the ulcer was completely cicatrized by the 12th January, and the patient was perfectly healthy.

Case II. Alexander Clapperton, ætat. six, was also seen by me on the 13th December. I found him sitting by the fire propped up with pillows; his face pale and bloodless, much swollen, particularly about the eyelids, and having a dull languid expression. His abdomen was tumid, the inferior extremities œdematous and hot on the surface, although the patient was exquisitely sensible to impressions of cold. His breathing was soft but hurried, and at times interrupted; and on auscultation the respiratory sound was much less distinct than usual. His pulse was small and rapid; tongue pale; breath fetid; urine scanty and high-coloured; and his stools dark and offensive. On inquiry I found that he had been complaining much for ten days, during the few last of which he had been unable to lie in bed; and that about six weeks previously he had had an attack of scarlatina, in which the rash had been perfect, and his recovery easy. I desired him to have some purgative medicine immediately, and then pills containing calomel and squills, one to be taken every seven or eight hours.

14th. Rather worse. Breathing more laborious; fluctuation perceptible in the abdomen; and the œdema of the inferior extremities so great as to render the integuments tense and shining. Frictions with the tartrate of antimony ointment to be employed over the abdomen and chest, the calomel and squills to be continued, and to have a solution of the supertartrate of potass for drink.

15th. There is no improvement. Prescriptions continued.

16th. Worse in every respect. Suffocation frequently threatened. Pulse excessively quick, and extinguishable on the slightest pressure. To have wine occasionally, and two grains of the sulphate of quinine repeated twice in the day.

17th. Died.

On inspection of the body two days after death, the following appearances were observed. Great bloodlessness of the whole body. Strong general adhesions in the chest. The right lung displaced, and much compressed, by about two pounds and a half of clear fluid. The lung itself more fleshy than natural, and on the tip of its superior lobe a considerable deposition of lymph, in the form of a yellow transparent jelly. The left lung more natural in appearance. The mucous membrane of the trachea and larger bronchia thickened, softened, and extremely vascular. The cellular substance of the anterior or mediastinum very much infiltrated with serum. A small quantity of serum in the pericardium. The heart pale, and containing in both ventricles large polypi. The abdomen exhibited evident marks of inflammatory action, with dropsical effusion to a considerable extent.

Case III. Agnes Clapperton, ætat. four, began to complain on the 17th December, the day of her brother's death. At first, the symptoms were languor, loss of appetite, costive bowels, tumid abdomen, œdematous legs, and scanty urine. In a few days these were succeeded by pyrexia, and afterwards by nearly the same dropsical appearances as have been described in the case of her brother, and also by the same termination on the 29th December. The treatment was somewhat different from that pursued in the former case. As the disease was seen from its commencement, bleeding and cathartics were resorted to on the first manifestation of febrile symptoms, and succeeded by the use of calomel and squills, the supertartrate of potass, a blister, and in the last stage, wine and the sulphate of quinine.

On dissection thirty hours after death, the appearances were these. In the thorax considerable adhesions; about twelve ounces of serum effused into the right side, and eight ounces into the left; great hepatization (the red hardening of Andral) of both lungs; the mucous membrane of the air-vessels very vascular; and the heart extremely pale, and containing in its right auricle a large polypus. In the abdomen, a very considerable quantity of fluid effused; the liver much enlarged; a large polypus in the vena hepatica; the gall-bladder very œdematous, there being nearly a quarter of an inch in distance between its coats; the covering of the kidneys thickened, but they themselves natural, with the exception of a very slightly speckled appearance externally, and being rather softer than usual internally; portions of the intestines very vascular, particularly near the termination of the ileum, where also there were several tubercles, and a slight ulceration; the mesenteric glands, especially in this neighbourhood, most extensively diseased, being of various sizes, from that of a pea to a walnut, some of them solid, others containing a fluid resembling light-coloured pus; and the glands of Peyer in many parts very large and excavated.

In perusing these cases, the reader may be inclined to think that the dropsies and the gangrenous sore may naturally be explained by a previous attack of scarlatina. In this way I accounted for the first two cases. But on learning from the parents of Agnes Clapperton that she had not had the slightest symptom of scarlet fever while Mary and Alexander had it, I was compelled to seek for another cause to resolve the mystery. For a week after A. C. was taken ill I could obtain no satisfaction. Although I could perceive that the family were in a state of extreme destitution, that every member of it had a most unhealthy aspect, yet my inquiries as to bad food were always met with an assurance that they had been living on nothing but what was perfectly wholesome. A neighbour of the family, to whom I next applied for information, hinted the probability of bad potatoes being the cause of the evil, as she knew for certain that the Clappertons had lately been subsisting almost entirely on such. I now renewed my efforts to ascertain the truth, and having directly charged the parents of the family with the use of this unwholesome diet, I obtained the following confession, apparently at much expense of feeling.

That for some time past the father had been out of employment, in consequence of which his family were left perfectly destitute; and, having too much pride to beg, and seeing starvation before them, they went to the fields, and gathered those potatoes which are exposed on the surface of the ground, and which are uniformly rejected by the farmer as utterly unfit for human use. That the potatoes in question were frosted,* were watery in consistence, some of them of a green, and others of a deep purple colour, and all of them having an excessively bitter taste. That in gathering these potatoes from the field, they often met with people similarly employed, but did not think that any of them did so for the purpose of human food, but were merely obtaining them for pigs; indeed, they had never heard of any one eating such potatoes. That this had been their aliment for upwards of six weeks previous to the beginning of December 1832, and that they had perhaps only a single meal of another kind once in eight days. That the potatoes had such a disagreeable taste as to be loathed, notwithstanding all the modes of preparation which their ingenuity could devise for rendering them more palatable. That in a very few days after using them, the whole family were seized with severe griping pains in the bowels, followed by diarrhœa of a green watery kind. That these bad effects continued with short intervals during the whole time that the potatoes were used, but that the children had not experienced them so severely or so constantly as the parents, which circumstance was accounted for by their occasionally getting a crust of bread from some of the neighbours.

To what extent the unwholesome food just described may have concurred with scarlatina in producing the effects detailed in Cases

* These potatoes, it may be necessary to explain, lie on the surface of the ground, and are exposed to the influence of the sun through the day, and to frost during night. Some conceive that frost effects a chemical change in the constituents of the potatoe-root, by converting its mucilage into sugar, from which acetic acid is speedily formed, and putrefaction induced. Others, again, consider that the watery part of the potatoe is converted into ice, which, occupying a larger space, separates the solid parts farther from each other, and produces, in consequence, a partial mechanical disorganization of structure.

I. and II. I am not prepared to say; but that a noxious influence must necessarily have been exerted by it on the constitutions of the patients, and consequently have disposed their systems to diseased action, particularly to that of dropsical effusion, I think is very obvious.

In the case of Agnes Clapperton, however, it seems exceedingly probable that the bad potatoes were the sole exciting cause of dropsy. Being aware that a dropsical tendency is often shown after very slight attacks of scarlatina, my inquiries in this case were repeated and strict; but I was uniformly and positively assured that, although A. C. laboured under slight diarrhœa in consequence of the potatoe diet still in use, while her brother and sister had the scarlet fever, she had neither sore throat, nor rash, nor any thing which could in the smallest degree indicate the existence of that disease in her person. The parents too could scarcely be deceived if any symptom of the fever had been present, having so simple and decisive a test to guide them, as comparison with the appearances exhibited by the other two children.

What gives additional strength to this opinion is the fact, that the same effect has often been produced by the same cause, namely, dropsy from frozen surface potatoes, in the case of horses and cattle. I have examined several individuals connected with farms in different districts of the country, and they all agree that feeding cattle or horses even for a very short time on such potatoes, unless plenty of *fodder* was given along with them, would inevitably generate dropsical effusion. In some districts, to avoid the dreaded evil, the cattle, being principally of the milch kind, are never allowed such food; the horses only get it in sparing quantities; but the swine, which are supposed incapable of being injured by vegetable productions, however rank and corrupt, are permitted them in an unrestricted abundance.

A Banffshire informant states, that in his part of the country cases of dropsy among the cattle in particular are very frequent, owing to the use of these potatoes, without a sufficient accompanying proportion of other food. He represents the deleterious influence of them on the animal to be shown, first, by the presence of griping, evinced by tossings of the head, movements of the legs, especially the hind ones, and rolling on the ground; secondly, by severe frothy purging; then by difficult respiration; and lastly, by serous effusion, which proves speedily fatal, if strong purgatives, diuretics, and the last resource, namely tapping, are not successful.

Striking points of analogy cannot fail to be observed between the facts mentioned in the above statement and those in the case of Agnes Clapperton. Previous to the dropsy, she had the griping and purging to a considerable extent, but certainly not by any means so severe as her father and mother; a circumstance which may perhaps explain the cause of their immunity from dropsical effusion; for it does not seem an unlikely supposition, that, had nature made the same efforts in her case to throw off the poisonous matter, she too might have escaped the fatal disease.

I may here mention, however, that her father had a most unhealthy, feeble appearance, and her mother looked even worse, had various complaints, and among the rest an abortion of a fourth months' pregnancy, all which ailments were not improperly attributable to her late unwholesome mode of living.

With regard to the manner in which the potatoes had acted in generating dropsy, and the appearances exhibited by dissection, I will not pretend to give a decided opinion.

Indigestible substances taken into the stomach, and improper modes of living, have by several authors been admitted as the cause of dropsies. Venables, in his Clinical Reports, p. 14, considers such causes as undeniable, and states a case of dropsy occurring from the use of fat bacon and cheese. Dr. Ayre, and several other authors, give cases both of hydrothorax, ascites, and anasarca, occurring separately, and in conjunction, from indulgence in what are called the pleasures of the table, and the immoderate use of fermenting liquors. In such cases as these, the disease must have originated in consequence of general debility, produced by the imperfect assimilation and conversion of aliment into chyle; by the scanty proportion of nourishing matter taken into the blood; and by the enfeebled condition of the whole vascular system from the existence of long-continued gastro-intestinal irritation. Now, though the unwholesome potatoes so often referred to might not give rise to dropsy, by any specific operation, they must have affected the system in the way that other indigestible substances would do.

The mass of disease found in the mesenteric glands of Agnes Clapperton may throw some light on the pathology of her case. It is well known that bad food is one of the great causes of such degenerations; and in her case, where the structure of the glands was so much diseased, it was impossible that the fluids could circulate freely; and perhaps the fluid resembling pus observed in so great quantity in some of the glands was in reality obstructed chyle. Dr. Ayre, p. 8, states a case of ascites proceeding from mesenteric disease, and remarks, that inflammatory action is propagated from the glands to the serous membrane investing them; "whence, as from a point, it gradually spreads along the membranous duplicature of the cavity." In the case of Agnes Clapperton, a similar diffusion of inflammatory action may have taken place.—*Edinburgh Med. and Surg. Journal.*

MISCELLANEOUS.

*Caspar Hauser. An Account of an Individual kept in a Dungeon, separated from all Communication with the World from early Childhood to about the Age of Seventeen.* Drawn up from Legal Documents. By Anselm von Feuerbach, President of one of the Bavarian Courts of Appeal. Translated from the German.

Many of our readers doubtless recollect to have seen, in the papers of the day, an account of an individual who it was represented had been found in Nuremberg, in a condition which left his previous life enveloped in the greatest mystery. This individual is the subject of the present short but extraordinary narrative, which may be truly termed a romance of real life. To the physiologist and metaphysician it is a most invaluable record, as shedding a new and most unforeseen light on some of the most intricate questions connected with the development of the human mind.

We shall not attempt to follow the history of this unfortunate being, except as connected with circumstances which fairly come within the design of a medical work. All that may be necessary to state at present is, that when Hauser was found, he appeared to be about sixteen or seventeen years of age, had never learned to speak, and that in all probability he had been shut out during his whole life from all communication with the world, in a narrow dark dungeon, in which he was always obliged to remain in a sitting posture, that his food had invariably been bread and water; in fact, that he had enjoyed a mere animal existence.

"Light had never shown upon this being, either on his eye or on his soul; and when he emerged from his lonesome darkness, he was like a new-born child in respect to all which must be acquired by experience, whilst the instruments for acquiring that experience, the natural faculties, of course differed from those of a child so far as they were affected by the mere age or growth of the individual."

His physical appearance when he appeared at Nuremberg is thus detailed. He was four feet nine inches in height, and about sixteen to seventeen years old. His chin and lips were very thinly covered with down; the dentes sapientiæ were wanting. His skin was fine and very fair; his limbs were delicate, and his feet showed no marks of having ever been confined by a shoe. His face was almost without expression, except that of a brutish obtuseness. He scarcely knew how to use his hands or fingers. His gait was like the first attempts of an infant to walk.

A medical examination was ordered to be made, the results of which, as stated by Dr. Osterhausen, in his report, present some curious anatomical detail. The patella, instead of forming a prominence, lay in a hollow. The tendons of the two vasti muscles were separately inserted into the tibia. From this peculiar formation, when Hauser sat down, with his leg and thigh extended horizontally on the floor, his body formed a right angle with the thigh, and the knee-joint was applied so closely to the floor as to prevent the passage of even a card beneath it.

He constantly evinced the strongest aversion to all kinds of meat and drink; and would only partake of bread and water. Even the smell of other food affected him disagreeably. The ingestion of the smallest quantity of wine, or coffee, occasioned him cold sweats and vomiting; even milk disordered his stomach. He was in possession of but two words with which he used to designate living beings; whatever appeared in the human form he called "bua," and to every animal he gave the name of "ross," (horse.) When first found, not only his mind, but most of his senses were in a state of torpor, but his nervous excitability was soon developed in an extraordinary degree; thus, the beating of a double drum near him threw him into convulsions. Not a spark of any religious belief was to be discerned in him.

The account which he gave of himself was as follows:

"He neither knows who he is nor where his home is. It was at Nuremberg that he first learnt that, besides himself and 'the man with whom he had always been,' there existed other men and creatures. As long as he can recollect, he had always lived in a hole, (a small low apartment, which he sometimes called a cage,) where he had always sat on the ground with bare feet, and clothed only with a shirt and pair of breeches. In his apartment he never heard a sound, whether produced by man, by an animal, or by any thing else. He never saw the heavens, nor did there ever appear a brightening (daylight) as at Nuremberg. Whenever he awoke from sleep he found a loaf of bread and a pitcher of water by him. Sometimes this water had a bad taste; whenever this was the case he could no longer keep his eyes open, but was compelled to fall asleep; and when afterwards he awoke, he found he had a clean shirt on, and that his nails had been cut. He never saw the face of the man who brought him his meat and drink. How long he continued to live in this situation he knew not."

It appears from his imperfect narrative, that his keeper once came behind him, so as not to be seen, and guided his hand so as to make it write something, and soon afterwards placed him on his feet, and attempted to teach him to walk, and finally, that the man carried him out from his prison on his back; of his journey Hauser knew but little, except that he fainted several times during it.

The state of his mind during his confinement, as far as could be ascertained, must have "been that of a human being immersed in his infancy in a profound sleep, in which he was not conscious even of a dream." In this condition he remained till he suddenly awoke, as it were, by the forcible impressions of external objects on his senses. He has no recollection of having ridden on his journey, though, from the deep sleep which riding always produces on him, this may have taken place. When he is asleep, noises appear to have no influence on him, and even rough treatment does not awaken him.

When first found, his saliva was as viscous as glue, but this afterwards became natural, on a change of food. He betrayed a great aversion to any glare of light, and his eyes were much inflamed. He was subject to spasmodic contractions on the left side of his body, especially when excited by any new object; or, when his mind was deeply engaged, these spasms were succeeded by a state of nervous rigidity. With regard to colours, Hauser always preferred the most glaring. At first he had no idea of size or distance; thus, when directed to look out of a window, he instantly drew back with repugnance; this he afterwards explained, by saying that it appeared as if the objects were close to his eyes. Drawings or paintings appeared to him as much relieved as if carved in wood.

It appears from a subsequent report of his medical attendant, that the multifarious impressions made upon his mind were too powerful, and produced a high state of nervous excitability.

"The muscles of his face were affected with frequent spasms. His hands trembled so much, that he was scarcely able to hold any thing. His eyes were inflamed, they could not bear the light. His hearing was so very sensible, that all loud speaking caused him violent pain."

He lost his appetite, and his gastric functions became disordered.

He was then removed to a quiet house, and no one permitted to see him. Here he for the first time slept in a bed, and began to dream. One of the most difficult undertakings was to accustom him to the use of ordinary food; this was a work of months; and it is a curious fact, that before he became accustomed to warm food, he was tormented with a constant thirst, drinking daily from ten to twelve quarts of water. After he became habituated to meat, his mental activity was diminished, his eyes lost their brilliancy and expression, and, in short, his state of morbid excitement gradually disappeared. This change of diet had also a remarkable effect on the development of his body, as he gained two inches in height in a very few weeks.

It would be impossible for us to detail the various mental phenomena that the gradual development of his faculties occasioned; we may, however, mention a few of the most striking, as exemplifying how slowly the mind acquires habits of just comparison. Thus, it was a long time before he could comprehend the difference between animate and inanimate objects, and he conceived that every motion which took place in any object was spontaneous; thus, if a sheet was blown down by the wind, he thought that it had run away. He supposed that a tree manifested its life by moving its branches and leaves, and that the rustling of the latter, when agitated by the wind, was the voice of the vegetable. To animals he for a long time ascribed the same as to men, and appeared to distinguish the one from the other only by the difference of their external form.

As to his sight, it was very peculiar, as he could see in the dark as perfectly as in the light; this was proved by numerous experiments: thus, in a perfectly dark night, he could distinguish blue from green, &c. His sight was also extremely acute, both as to distance and nicety. His hearing was as remarkable. But of all his senses, that which was the most troublesome to him, and which rendered his life miserable, was that of smell. What to all other persons was entirely senseless, was not so to him. The odour of a rose overcame him; in fact, every smell except those of bread, fennel, anise and carraway, were more or less disagreeable to him. He could distinguish fruit trees from each other at a considerable distance by the smell of their leaves. The odour of old cheese made him feel unwell, and caused vomiting. What are considered by most persons as unpleasant smells, were much less offensive to him than what we consider as agreeable perfumes.

There is a fact connected with this acuteness of smell, which settles the question of the emanations from burial-grounds; when he walked near a churchyard, the smell from it, which was not perceptible to any one else, occasioned him a complete paroxysm of fever.

One of the most extraordinary peculiarities of Hauser's temperament was his susceptibility to magnetic and metallic excitements. A toy was given him furnished with a magnet; this he would not use, as he complained it occasioned him unpleasant sensations. On learning this fact, Professor Daumer made some experiments on him with a magnet. When the north pole was presented to him, he complained of pain in his stomach, and that a current of air appeared to flow from him towards the magnet; the south pole had less effect on him. These experiments were varied as much as possible, but he was never deceived. In respect to his sensibility of the presence of metals, and his ability to distinguish them by his feelings, many instances are adduced. He described his sensations of the contact of metals, as a feeling of drawing or attraction, which passed over him, accompanied at the same time with a chill, which ascended, according as the objects were different, more or less up the arm. This sensibility he gradually lost. When he caught a cat by the tail, he was seized with a strong fit of shivering, and felt as if he had received a blow upon the hand.

His present state is thus described.

" His mode of life is that which is common to most men. With the exception of pork, he eats all kinds of meat, if not seasoned with hot spices. His favourite condiments still are carraway, fennel, and coriander. His drink continues to be water, and only in the morning he takes a cup of unspiced chocolate instead of it. All fermented liquors, beer and wine, as also tea and coffee, are still an abomination to him; and, if a few drops of them were forced upon him, they would infallibly make him sick.

"The extraordinary, almost preternatural elevation of his senses, has also been diminished, and has almost sunk to the common level. He is indeed still able to see in the dark; so that, in respect to him, there exists no real night, but only twilight; but he is no longer able to read in the dark, nor to recognise the most minute objects in the dark at a great distance; whereas he was formerly able to see much better and more distinctly in a dark night than by day-light; the contrary is now the case. Like other men, he is now able to bear and he loves the light of the sun, which no longer distresses his eyes. Of the gigantic powers of his memory and other astonishing qualities, not a trace remains. He no longer retains any thing that is extraordinary, except his wonderful fate, and the gentleness of his disposition."

In the above abstract we feel that we have not given as connected a view of the operations of Hauser's feelings and mental faculties as they were gradually acted on, and developed by the action of external objects, as could have been wished; this however our limits prevented, and we must therefore refer such of our readers as wish for more information, to the work itself, which will amply repay an attentive perusal.—*American Journal of Med. Sciences.*

---

### MATERIA MEDICA.

*Influence of Vinegar in lessening the Urinary Secretion.* Mr. John Dalton, in a course of experiments for comparing the quantity of food taken with that of the different secretions, found on one day a great defalcation in the amount of urine which he passed, and for which he could discover no cause, unless his having taken at dinner-time a teaspoonful or two of vinegar, could account for it. To be satisfied of this, he took, some days after, an ounce of vinegar in four equal portions during one day; and the effect was a very great diminution of urine on that day, amounting to fifteen ounces less than the average quantity, ($48\frac{1}{2}$ oz.) Mr. D. says that there did not appear to be any increased effect in any other secretion, as a compensation for this diminution.—*Memoirs of the Lit. and Philosophical Society of Manchester, Vol. V., N.S.* 1831.

---

